# Lumboperitoneal Shunt Malfunction Due to Misplacement of the Lumbar Catheter Into the Spinal Subdural Extra-arachnoid Space: A Case Report

**DOI:** 10.7759/cureus.92006

**Published:** 2025-09-10

**Authors:** Kohei Hashida, Tatsuya Tanaka, Shuhei Yamazaki, Ryohei Sashida, Akira Matsuno

**Affiliations:** 1 Department of Neurosurgery, International University of Health and Welfare Narita Hospital, Narita, JPN

**Keywords:** idiopathic normal pressure hydrocephalus, lumboperitoneal shunt, shunt malfunction, shuntography, spinal subdural extra-arachnoid space

## Abstract

Lumboperitoneal (LP) shunting is an established treatment for idiopathic normal pressure hydrocephalus (iNPH). Although generally considered less invasive, LP shunts carry the risk of lumbar catheter malposition, including rare misplacement into the spinal subdural extra-arachnoid space (SSES), a potential cause of shunt malfunction.

A 72-year-old woman presented with gait disturbance, cognitive decline, and urinary incontinence. Magnetic resonance imaging (MRI) revealed ventriculomegaly and disproportionately enlarged subarachnoid space (DESH). Her symptoms improved after a tap test, and an LP shunt was placed under fluoroscopy using a CERTAS™ Plus programmable valve. Initial postoperative improvement was observed. However, her symptoms worsened six months later despite normal imaging and valve adjustments. Shuntography revealed localized contrast pooling along the thoracolumbar nerve roots without spinal canal spread. Computed tomography (CT) confirmed catheter placement in the SSES. Revision surgery using the one-piece method with fluoroscopic guidance achieved accurate catheter repositioning. The patient's symptoms subsequently improved.

SSES is a potential but often unrecognized anatomical space created between the dura and arachnoid mater during puncture. Although the cerebrospinal fluid (CSF) may drain initially, the lack of communication with the subarachnoid space can result in delayed shunt failure. Diagnosis requires imaging, and initial clinical improvement may be misleading. The one-piece method enables safe, precise catheter reinsertion and avoids reconnection errors. Clinicians should consider catheter misplacement into SSES as a cause of LP shunt failure, even when early postoperative improvement is observed. Fluoroscopy-guided placement and vigilant postoperative follow-up are essential for optimal outcomes.

## Introduction

Idiopathic normal pressure hydrocephalus (iNPH) is a reversible clinical syndrome characterized by a classic triad of gait disturbance, cognitive impairment, and urinary incontinence. It significantly impairs activities of daily living (ADL) and quality of life (QOL) in the elderly population. With the progression of a super-aged society, the number of patients with iNPH is expected to increase in the future [1,2].

Standard surgical treatments for iNPH include ventriculoperitoneal (VP) shunting and, more recently, lumboperitoneal (LP) shunting, which has been increasingly adopted. A prospective study (SINPHONI-2 trial) demonstrated comparable efficacy between VP and LP shunting [1,2]. While LP shunting is considered a less invasive option as it avoids cranial surgery, it involves lumbar catheter insertion, which carries procedure-specific risks, such as difficulty in puncture, catheter rupture, delayed disconnection, and misplacement [1-6].

Among these, although rare, misplacement of the lumbar catheter into the spinal subdural extra-arachnoid space (SSES), a potential space between the dura mater and arachnoid membrane, can occur and result in shunt malfunction [6]. The SSES is a dissectible space that does not normally exist but can be created by puncture-related trauma. In the field of anesthesiology, inadvertent delivery of anesthetic agents into this space during epidural or nerve root blocks has been associated with unexpectedly extensive neural blockade or respiratory depression [7-9]. However, reports of SSES misplacement during LP shunt procedures in the field of neurosurgery remain extremely limited [6].

Here, we report a rare case in which an LP shunt catheter was inadvertently inserted into the SSES, leading to temporary postoperative symptom improvement followed by clinical deterioration. The diagnosis was established using shuntography and computed tomography (CT), and reoperation was successfully performed using fluoroscopy and a peel-away sheath via the “one-piece method” [10]. We present this case along with a literature review to highlight diagnostic and therapeutic considerations for SSES misplacement in LP shunting.

## Case presentation

**Figure 1:**
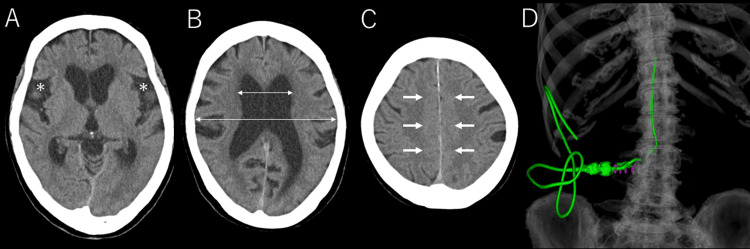
Characteristic head CT findings of idiopathic normal pressure hydrocephalus and LP shunt placement CT: computed tomography; LP: lumboperitoneal Head CT demonstrates typical findings of disproportionately enlarged subarachnoid space hydrocephalus (DESH) associated with idiopathic normal pressure hydrocephalus, including widening of the Sylvian fissures (A, asterisk), ventricular enlargement with an Evans index of 0.34 (B, double arrows), and narrowing of the high convexity and midline sulci (C, arrows). A 3D CT image confirms the placement of the LP shunt (D)

A 72-year-old woman presented with cognitive decline and urinary incontinence. Her Mini-Mental State Examination-Japanese (MMSE-J) score was 20 out of a maximum of 30 points, and the three-meter Timed Up and Go (TUG) test result was 12.65 seconds. The total score on the idiopathic normal pressure hydrocephalus grading scale (iNPHGS) was 8, with gait disturbance (g) of 2, cognitive impairment (c) of 3, and urinary disturbance (u) of 3 [1]. Head CT revealed ventricular enlargement with an Evans index of 0.34 and imaging features characteristic of disproportionately enlarged subarachnoid space hydrocephalus (DESH), including widened Sylvian fissures and tight high-convexity sulci (Figures 1A-1C).

A tap test temporarily improved the symptoms, with MMSE improving from 20 to 23, TUG from 12.65 to 9.87 seconds, and iNPHGS from 8 to 7. Based on these findings, the patient was diagnosed with iNPH. An LP shunt procedure was performed under fluoroscopic guidance using a CERTAS™ Plus programmable valve (Integra LifeSciences Corporation, USA) (Figure 1D). A paramedian approach was used to insert a Tuohy needle, and cerebrospinal fluid (CSF) outflow was confirmed. A lumbar catheter was inserted without resistance, and CSF outflow was also observed from the catheter tip. Fluoroscopic guidance during lumbar catheter insertion was performed intermittently rather than continuously.

Postoperatively, the patient showed improvement. At one week after surgery, MMSE-J improved to 25, TUG to 9.54 seconds, and iNPHGS to 4 (g1, c1, u2). However, symptoms recurred at six months postoperatively, and iNPHGS worsened to 6 (g2, c3, u3). Although a shunt malfunction was suspected, brain CT, plain X-ray of the shunt system, and the pumping test were all normal. Changing the valve setting from level 4 to level 3 did not improve the symptoms.

**Figure 2:**
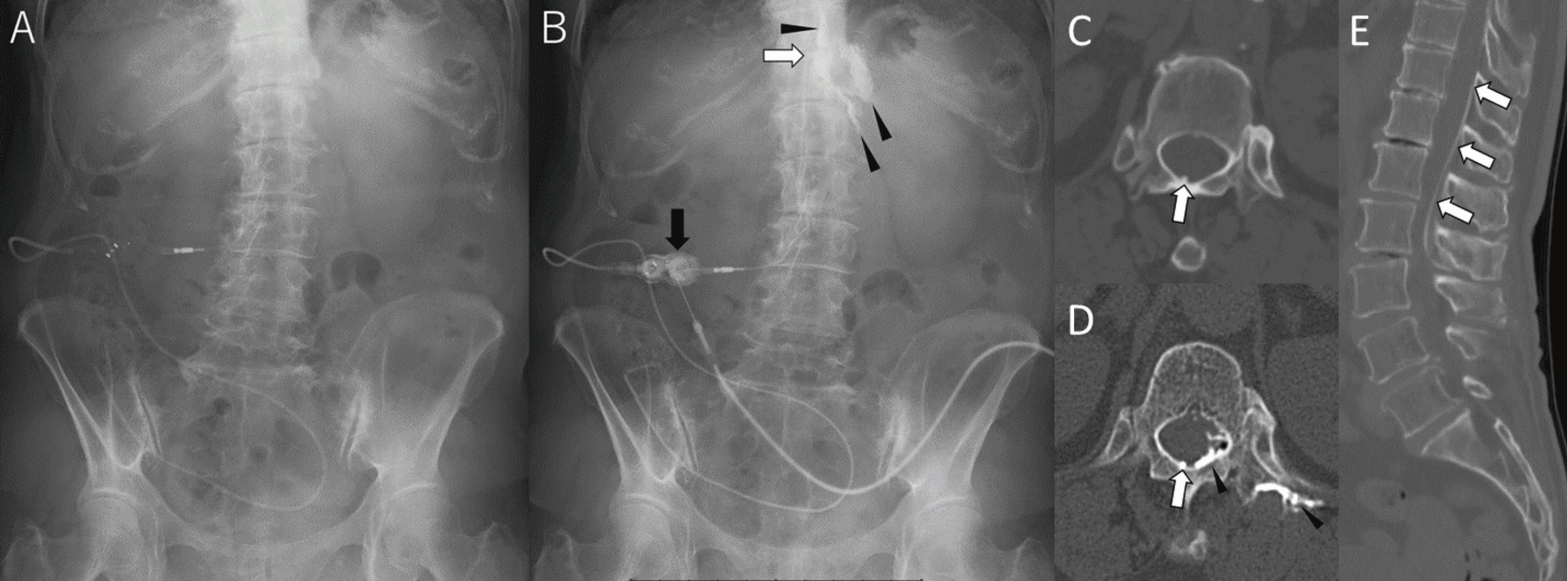
Shuntography findings LP: lumboperitoneal; CT: computed tomography (A) The LP shunt appears to be normally positioned on plain radiography. (B) After puncturing the reservoir with a 23 G needle and injecting contrast medium (black arrow), the contrast travels through both the lumbar and peritoneal catheters but fails to diffuse into the spinal canal. Instead, localized contrast accumulation is observed along the right T11–T12 nerve roots (arrowhead). (C) Axial CT image at the T12 level shows the catheter tip positioned within the spinal canal but adjacent to its dorsolateral wall. (D) Contrast is distributed along the dorsolateral subdural extra-arachnoid space and nerve roots, without entry into the subarachnoid space. (E) Sagittal CT reconstruction further confirms the dorsolateral position of the catheter tip within the spinal canal

At 13 months postoperatively, a shuntography was performed. A 23 G needle was used to puncture the reservoir, but no CSF reflux was obtained. Iohexol, a nonionic water-soluble iodinated contrast agent (Iopamiron 240 mg I/mL, Bayer, USA), was injected. The contrast passed through both the lumbar and peritoneal catheters without obstruction, but no intrathecal spread was observed. Instead, focal contrast accumulation was noted along the right T11-T12 nerve roots (Figures 2A, 2B).

Subsequent CT demonstrated contrast retention within the SSES, and the tip of the lumbar catheter was located within this compartment. Axial and sagittal CT reconstructions clearly demonstrated that the catheter tip was positioned within the spinal canal but adjacent to its dorsolateral wall (Figures 2C-2E). These findings indicated that the initial lumbar catheter had been mistakenly inserted dorsally into the SSES through the dura.

**Figure 3:**
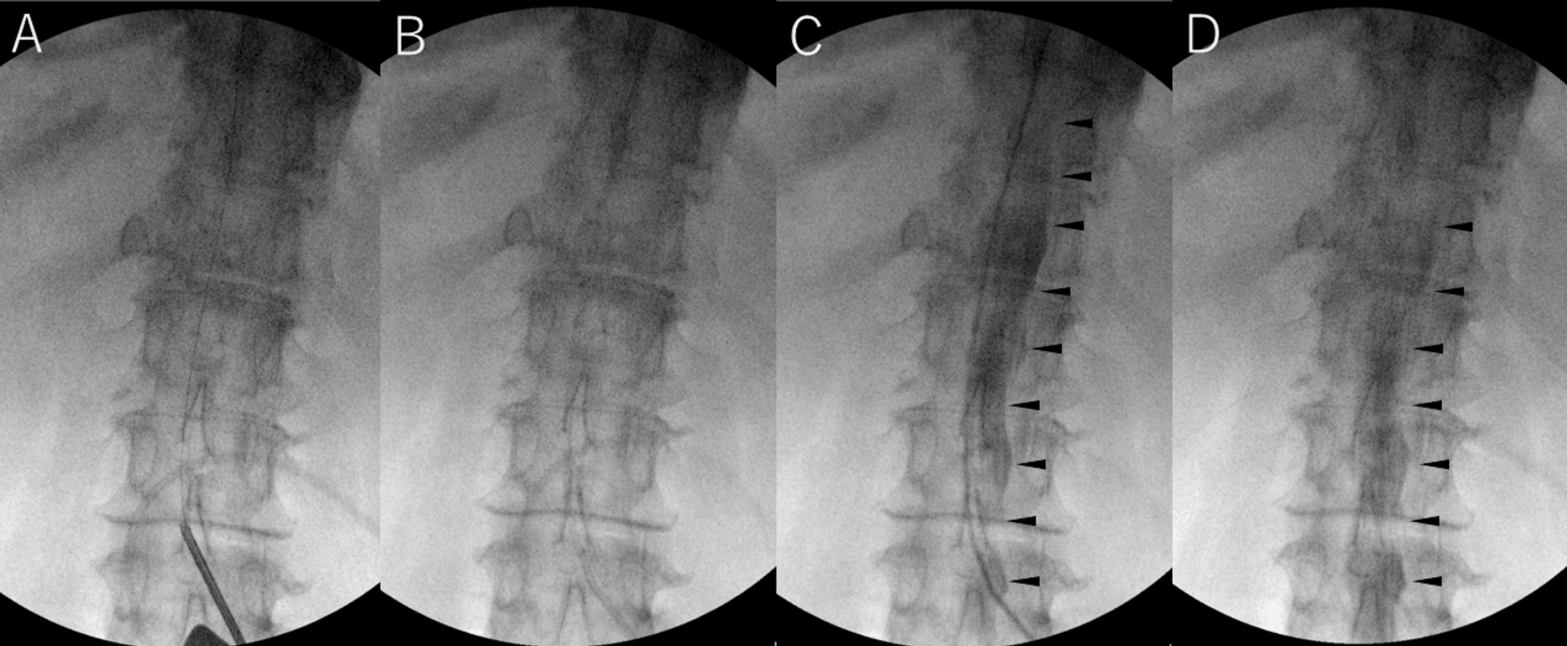
Insertion of the lumbar catheter using the one-piece method Insertion of the lumbar catheter using the one-piece method. (A) A 14 G Tuohy needle was inserted into the L2/3 interlaminar space via a paramedian approach, and a 0.032-inch guidewire was advanced under fluoroscopic guidance. (B) The Tuohy needle was withdrawn, and a 5 Fr peel-away sheath was inserted along the guidewire. The dilator and guidewire were then removed. (C) Three milliliters of contrast were injected, confirming its spread into the subarachnoid space (arrowhead). (D) The catheter was inserted through the sheath, and proper positioning was verified fluoroscopically before removing the sheath

As the misplacement was deemed the cause of the shunt malfunction, a revision surgery was scheduled. Under local anesthesia, the previous dorsal incision was reused to expose the lumbar catheter, which was removed from the subdural space without resistance. The catheter was then reinserted under fluoroscopy using the following technique:

A 14 G Tuohy needle was inserted into the L2-3 interspace via a paramedian approach, and a 0.032-inch guidewire was advanced under fluoroscopy (Figure 3A). The Tuohy needle was withdrawn, and a 5 Fr peel-away sheath was introduced over the guidewire. After removing the dilator and guidewire, CSF outflow from the sheath was confirmed, and 3 mL of iohexol was injected to verify subarachnoid spread (Figures 3B, 3C). A new catheter was inserted through the sheath and positioned under fluoroscopic guidance, after which the sheath was removed (Figure 3D). This reinsertion was performed using the “one-piece method,” in which the lumbar catheter, valve, and peritoneal catheter are preassembled as a single unit to allow smooth tunneling and reduce the risk of disconnection or malposition.

**Figure 4:**
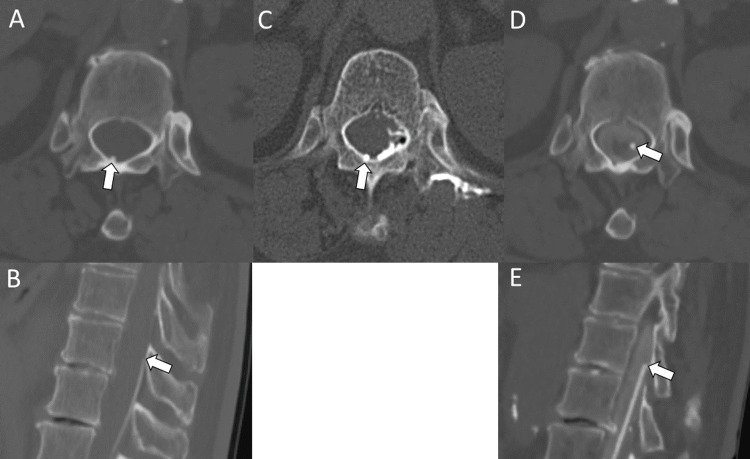
Lumbar catheter tip position before and after revision CT: computed tomography (A) Axial view and (B) sagittal view: The initial postoperative CT shows the catheter tip located at the dorsolateral aspect of the spinal canal (arrow). (C) CT at the time of shuntography also demonstrates the tip positioned at the dorsolateral aspect of the canal (arrow). (D) Axial view and (E) sagittal view: postrevision myelographic CT shows appropriate contrast spread within the subarachnoid space and the catheter tip abutting the spinal cord (arrow)

Postoperative CT confirmed that the catheter was correctly positioned in the subarachnoid space and that the contrast had appropriately spread into the subarachnoid space (Figure 4).

At four weeks postoperatively, MMSE-J, TUG, and iNPHGS scores showed improvement to 19 points, 9.75 seconds, and 6 (g1, c3, u2), respectively. This case report was approved by the Institutional Review Board of the International University of Health and Welfare (approval number: 23-Nr-010). Written informed consent was obtained from the patient for the publication of this case report and the accompanying images.

## Discussion

This case presents a rare instance of LP shunt malfunction caused by misplacement of the lumbar catheter into the SSES, where the patient exhibited transient postoperative improvement followed by symptom recurrence.

LP shunting is one of the standard treatments for iNPH, and its effectiveness has been widely reported in recent years [1,2]. However, unlike VP shunting, LP shunting involves lumbar catheter insertion, which carries specific procedural risks-particularly when performed without image guidance. These include misplacement, kinking, subcutaneous migration, and fracture [3,4,6]. Tanaka et al. reported catheter malposition in 15.3% of 72 cases of LP shunting performed without fluoroscopy, highlighting the importance of intraoperative visualization [3].

The SSES is an anatomically virtual space between the tightly adherent dura mater and arachnoid membrane, but it may be created by needle trauma or mechanical dissection during puncture [6-9]. When a catheter is inadvertently placed in the SSES, CSF outflow may still occur, but communication with the subarachnoid space is absent, resulting in shunt malfunction. Izutsu et al. reported two cases of shunt failure caused by SSES misplacement, in which shuntography and CT were essential for diagnosis [6]. Based on their findings and the present case, a lumbar catheter tip located laterally within the spinal canal on axial CT should raise suspicion for SSES misplacement.

Interestingly, some cases, including the present one, have demonstrated transient clinical improvement after SSES misplacement [6]. Izutsu et al. proposed two hypotheses to explain this phenomenon [6]: (1) a transient CSF leak caused by arachnoid tear during puncture may have reduced ventricular pressure; and (2) spontaneous re-adhesion of the arachnoid and dura over several weeks to months may have led to delayed obstruction and shunt failure. In addition, transient improvement may also be attributable to temporary decompression of the nerve root sleeves due to CSF leakage, which could partially alleviate symptoms until re-adhesion occurs. These findings emphasize the need for serial clinical assessment rather than over-reliance on initial postoperative improvement.

In this case, revision surgery was performed using the “one-piece method” with a peel-away sheath and fluoroscopic guidance to reinsert the lumbar catheter [10]. The “one-piece method” refers to reinsertion of the LP shunt as a preassembled single unit consisting of the lumbar catheter, valve, and peritoneal catheter. This approach minimizes intraoperative manipulation, reconnection errors, and catheter displacement [10]. The patient experienced symptomatic improvement after accurate catheter placement using this technique.

To prevent catheter misplacement into the SSES during LP shunt procedures, intraoperative fluoroscopy and shuntography should be actively employed. In cases of postoperative symptom recurrence, physicians must consider the possibility of SSES misplacement as part of the diagnostic approach. Accurate diagnosis was achieved via shuntography and CT. Furthermore, surgical revision using reliable techniques such as the one-piece method can ensure accurate and safe reinsertion.

## Conclusions

This case highlights a rare instance of LP shunt malfunction due to lumbar catheter misplacement into the SSES. Accurate diagnosis was achieved via shuntography and CT. Revision using the one-piece method under fluoroscopy enabled safe and precise catheter reinsertion. Even with transient postoperative improvement, catheter misplacement should be considered, particularly when symptoms recur despite normal routine imaging, and early evaluation is essential for timely management.
